# Evolution of the Teaching of Animal Welfare Science, Ethics and Law in European Veterinary Schools (2012–2019)

**DOI:** 10.3390/ani10071238

**Published:** 2020-07-21

**Authors:** Nancy De Briyne, Jovana Vidović, David B. Morton, Manuel Magalhães-Sant’Ana

**Affiliations:** 1 Federation of Veterinarians of Europe, 12B-1040 Brussels, Belgium; 2 Department of Veterinary Medicine, Faculty of Agriculture, University of Novi Sad, Trg Dositeja Obradovića 8, 21000 Novi Sad, Serbia; jovanavidovic21@gmail.com; 3 School of Biosciences, University of Birmingham, Birmingham B15 2TT, UK; dbmgm2@gmail.com; 4 CIISA—Centre for Interdisciplinary Research in Animal Health, Faculty of Veterinary Medicine, University of Lisbon, 1300-477 Lisboa, Portugal; mdsantana@gmail.com; 5 Ordem dos Medicos Veterinários, Av. Filipe Folque, 10J, 4° Dto., 1050-113 Lisboa, Portugal

**Keywords:** animal welfare science, ethics, law, model curriculum, Day-1 competences, veterinary education, veterinary faculty, veterinary ethics, animal ethics

## Abstract

**Simple Summary:**

Nowadays, animal welfare is seen as a ‘common good’ and a societal expectation. Veterinarians are expected to promote and ensure the welfare of animals under their care by using their scientific knowledge and skills in ethical reasoning and advocacy. In 2013, the Federation of Veterinarians of Europe (FVE) and the European Association of Establishments for Veterinary Education (EAEVE) adopted the Day-1 competences on animal welfare science, ethics and law for veterinary undergraduate education after having surveyed 33 European veterinary schools in 2012. In 2019, a follow-up survey was done to monitor the evolution of animal welfare teaching in Europe. A total of 82 responses were received, representing 57 veterinary schools from 25 European countries. Overall results showed that the teaching of animal welfare science, ethics and law has increased in response to growing societal needs, and that welfare is more and more internally embedded in the profession, which is reflected in the curriculum.

**Abstract:**

Nowadays, animal welfare is seen as a ‘common good’ and a societal expectation. Veterinarians are expected to promote and ensure the welfare of animals under their care by using their scientific knowledge and skills in ethical reasoning and advocacy. Veterinary education must equip veterinary graduates with the necessary competences to fulfil these roles. In 2013, the Federation of Veterinarians of Europe (FVE) and the European Association of Establishment of Veterinary Education (EAEVE) adopted the Day-1 competences on animal welfare science, ethics and law for veterinary undergraduate education after having surveyed European veterinary schools in 2012. In 2019, the FVE carried out a follow-up survey to monitor the evolution of animal welfare teaching in Europe. A total of 82 responses were received, representing 57 faculties from 25 European countries. Overall results showed that the teaching of animal welfare science, ethics and law has increased in response to growing societal needs, and that welfare is more and more internally embedded in the profession, which is reflected in the curriculum. Nevertheless, at least one quarter of European schools still only partially meet the 2013 Day-1 competencies. This indicates the need for greater efforts, both from the EAEVE and from individual schools, to ensure that the teaching of animal welfare across Europe is standardised.

## 1. Introduction

Nowadays, animal welfare is seen as a ‘common good’ and, as such, as a shared responsibility and an ethical obligation [1]. It matters to humans as much as to the animals themselves, and is a key concept for reaching the United Nations sustainable development goals [1,2]. Animal welfare is a multidimensional concept, composed of scientific, ethical and legal dimensions [3]. Veterinarians do, and should, play a key role in promoting animal welfare by virtue of their scientific knowledge of animals’ wants and needs, their skills in ethical reasoning and decision-making, and their advocacy within the legal framework [4,5]. To fulfil this role in promoting animal welfare, it is of the utmost importance that veterinary students, during their undergraduate studies, receive robust education on animal welfare science, ethics and law, including practical teaching and clear explanations of how this interacts with other veterinary fields such as genetics, animal health and food safety.

In 2012, given on-going scientific developments and society’s expectations in this field, the Federation of Veterinarians of Europe (FVE) partnered with the European Association of Establishments for Veterinary Education (EAEVE), which is the official accreditation authority for veterinary education establishments within Europe, to examine animal welfare in the context of European veterinary education. In 2013, both associations agreed on a model curriculum in animal welfare science, ethics and law (hereafter AWSEL) [6]. That document adopted six Day-1 competences in AWSEL, expressed in terms of 34 learning objectives (Table 1). This 2013 model curriculum is part of the EAEVE/FVE evaluation and accreditation system which ensures that the agreed upon benchmark educational levels are met. 

In order to inform these Day-1 competences, two surveys were conducted in 2012. First, through the Animal WelfAre Research in an enlarged Europe (AWARE) Project, a survey was conducted to map the under- and post-graduate teaching of animal welfare in veterinary and animal science faculties in 26 European countries [7]. This first survey identified considerable regional differences in terms of the amount of teaching and pedagogical approaches. It also found indications that animal welfare teaching in Europe was increasing [7]. Next in 2012, a second survey was carried out to specifically evaluate the teaching practices in AWSEL and the support and feasibility of the draft model curriculum in European undergraduate veterinary education. This second survey which was answered by 33 veterinary faculties across Europe showed general support for the draft model curriculum. Fifty-five percent of schools did not meet the suggested model curriculum, with most respondents saying they could meet it within 5 years (unpublished data). Lack of space in the curriculum and limited financial resources (namely insufficient resources to recruit AWSEL staff) were the main impediments to implementing the AWSEL curriculum.

Seven years later, in 2019, a follow-up survey was done to assess the progress of AWSEL teaching in European veterinary schools, including whether the 2013 Model Curriculum was still able to cover the important Day-1 competences in animal welfare. This paper reports the findings from this survey and provides further reflections on the evolution of AWSEL education in Europe.

## 2. Materials and Methods

The Federation of Veterinarians of Europe (FVE) developed and launched a semiquantitative (multiple choice and open answer) online survey to collect data on the teaching of AWSEL in European veterinary schools (see Appendix A). The survey was written in English and was based on the 2012 (second) survey, with some additions and changes, namely regarding the 2013 Day-1 competencies. The survey looked into the AWSEL curriculum, i.e., whether there were mandatory or elective subjects, the number of contact hours and how the teaching of AWSEL compared with that of other contemporary subjects, defined as emergent areas in veterinary science education (Ranked from 1 to 5). The survey was sent by email to the deans of all 96 European Veterinary Education Establishments (VEEs) from the 34 countries which are members of the EAEVE (list available at www.eaeve.org/about-eaeve/members.html). It was also sent to the Diplomats of the European College of Animal Welfare and Behavioural Medicine (ECAWBM), many of whom teach animal welfare at veterinary schools. Prospective respondents were instructed to forward the survey to the person in their institution who would be best qualified to complete it. The respondents were informed that the survey aimed to provide an overview of the current situation in terms of AWSEL teaching in Europe, and that its findings would be made public without mentioning respondents or teaching establishments. Respondents were allowed to defer answers, and not all respondents answered all questions. The survey was launched on 15 October 2019 through SurveyMonkey, and was open for four months. For 12 veterinary schools, more than one answer was received, in which case the answers were compared, and any discrepancies were noted (the case for 4 VEEs) and double checked with the respondents. As a quality assurance measure, some teaching hours were also double checked with the schools’ Self Evaluation Reports, published on the EAEVE website. Data were handled and descriptive statistical analyses were performed using Microsoft Excel, including a comparative analysis between the 2012 and the 2019 surveys.

## 3. Results

A total of 82 responses were received. Four answers came from VEEs outside Europe (Japan) and were excluded. The 78 remaining answers represented 57 VEEs (59% response rate) from 25 countries throughout Europe. For 12 VEEs, more than one reply was received. Of the 57 VEEs, 49 VEEs were located in EU/EFTA countries and 8 were outside EU/EFTA countries. Fourteen VEEs answered both the 2012 and 2019 surveys (Table 2). This section describes quantitative results only. The results from open-ended questions were imbedded into the discussion.

### 3.1. Progress of Animal Welfare Science, Ethics and Law (AWSEL) Education

**Figure 1:**
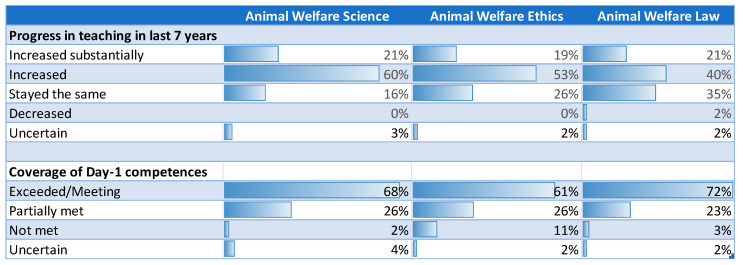
Progress in teaching animal welfare science, ethics and law within the last seven years (2012–2019) and percentage of European veterinary schools regarding coverage of Day-1 Competences in animal welfare science, ethics and law.

In terms of the time spent teaching AWSEL in undergraduate veterinary studies within the last 6 years, 81% (N = 46) replied that it had increased or increased substantially for animal welfare science (AWS), 72% (N = 59) for animal welfare ethics (AWE) and 61% (N = 50) for animal welfare law (AWL). The time spent teaching AWSEL has not decreased except for AWL at one school (2%) (Figure 1).

### 3.2. Relevance of the 2013 EAEVE/FVE Model Curriculum

Ninety-three percent of VEE respondents (N = 53) considered that the current relevant learning objectives and Day-1 competences in AWSEL were still being covered by the 2013 EAEVE/FVE model curriculum. Five percent disagreed (N = 3), and 2% (N = 1) were unsure. Nonetheless, suggestions were made to improve the EAEVE/FVE model curriculum (cf. discussion), namely to add the following topics: recognising and dealing with animal abuse; veterinary duties concerning the Official Controls Regulation (Reg. (EU) 2017/625); and the application of the Three Rs in the protection of animals used for scientific purposes.

In terms of the coverage of the Day-1 competencies in AWSEL at each school (Figure 1), the majority of respondents replied that they covered or even exceeded the Day-1 competences for AWS (68%; N = 39), for AWE (61%; N = 35) and for AWL (72%, N = 42). Figure 1 also shows the percentages of those who met them partially or not at all.

Most (63%) VEEs that did not yet meet all Day-1 competences in 2019 felt that they could meet them within 1 to 3 years, while the remaining participants felt that 4–5 years were needed. The main obstacles quoted were related to lack of space in the curriculum (57%) and a lack of qualified teachers (43%).

### 3.3. Importance of AWSEL Within the Curriculum

Respondents were asked whether AWSEL is part of the core obligatory curriculum, i.e., whether students have exams on the subject and can pass or fail. This was the case in almost all veterinary schools, except one (2%) for AWS, six (11%) for AWE and four (7%) for AWL.

For 74% of the VEEs (N = 42), animal welfare is an independent study topic, similarly to other subjects, such as surgery or radiology. Seven percent (N = 4) considered that AWSEL is not emphasised as much as other study subjects, and 17% (N = 10) answered that it is taught as part of other subjects. Some VEEs (N = 18) offer additional AWSEL-related elective courses (e.g., fish welfare, MOOC on animal experimentation, pharmacological aspects of pain therapy or kennel welfare), while others (N = 15) embed it in a mentored independent study (e.g., in an elective project or deeper study thesis).

### 3.4. Contact Hours of AWSEL Teaching

**Figure 2:**
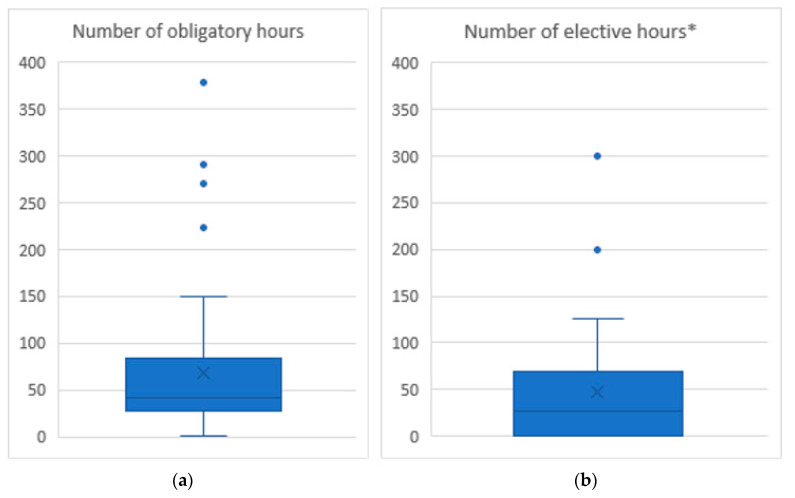
The number of (**a**) obligatory and (**b**) elective hours delegated to animal welfare studies within the curriculum (boxes indicate the second and the third quartiles; bars denote the median; x denotes the average value, whiskers indicate the 5th and 95th centiles; dots denote outliers). *Data available for only 30 VEEs, as not all had electives.

The number of hours spent teaching AWSEL varied significantly depending on the VEE, as shown in Figure 2. The average obligatory teaching of AWSEL was 73 h (median 41 h). Only 30 VEEs provided data on electives, with an average of 47 and a median of 27 h.

### 3.5. Importance of AWSEL Within the Faculty in Comparison to Other Subjects

**Figure 3:**
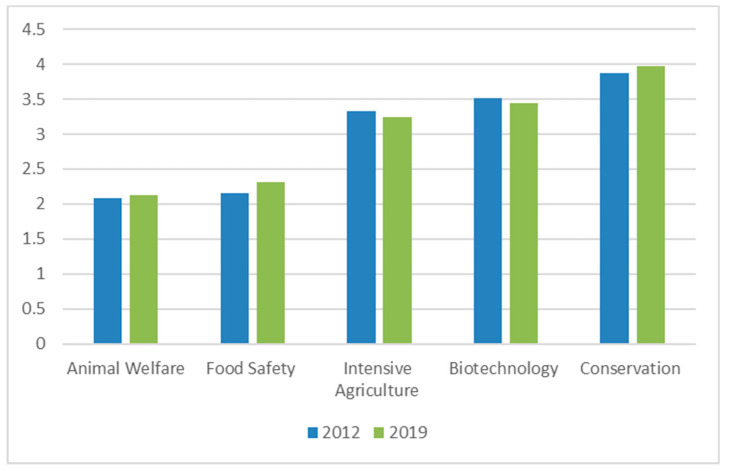
Ranking subjects (average value) according to importance in veterinary education (1—most important; 5—least important).

Animal welfare (AWSEL) was ranked as an important subject, i.e., above food safety, intensive agriculture, biotechnology and conservation. This was very similar to the ranking in 2012 (Figure 3).

When asked to choose between animal welfare and antimicrobial resistance as the most important topic, 51% choose the former and 49% the latter. Most respondents (60%; N = 34) stated that knowledge of AWSEL is viewed as a very important subject in their veterinary college, 37% (N = 21) answered that it is somewhat important, one respondent considered it not important and one was unsure of its importance. On the question of the importance attached to having a course entitled ‘animal welfare’ in the veterinary undergraduate curriculum, 79% (N = 45) considered this very important, 16% (N = 9) considered it important and 5% (N = 3) considered it to be irrelevant or not important.

### 3.6. The Teaching of Different Aspects of Animal Welfare as Day-1 Competences

Respondents were asked about the teaching of different topics in animal welfare science, and the answers were compared with those of 2012. This showed that teaching of this discipline has substantially increased in the last 7 years, e.g., animal welfare assessment of wild animals was only taught by 24% in 2012 versus by 51% in 2019; and the promotion of positive animal welfare increased from 59% to 98% (see Table 3).

## 4. Discussion

This paper described the progress made in Europe within the last seven years in terms of animal welfare science, ethics and law in veterinary undergraduate education, through a semiquantitative online survey. Although some countries were regrettably not represented (such as Austria and Hungary), we were able to collect responses from schools that represent the entire European geographic spectrum. There are both societal and professional expectations for veterinarians to provide leadership in AWSEL through actions that stimulate and contribute to public discourse, that build community trust and that support community consensus regarding appropriate animal protection, use, care and treatment [4,5,8]. A large-scale European study showed that European citizens attach great importance to animal welfare, want more information on the conditions in which farm animals are treated, and feel the EU should promote greater awareness of animal welfare at a global level [9]. Veterinarians should also share knowledge to promote and support welfare-focused animal care standards and practices [4,10].

To help address these demands, in 2013 a model curriculum was devised between EAEVE, the umbrella body of, at the time, 96 European VEEs, and the FVE, the umbrella body of 44 veterinary associations from 39 European countries [6]. This 2013 model curriculum is part of the EAEVE/FVE evaluation and accreditation system (itself accredited by ENQA [11]) which has been running for well over a decade to ensure that veterinary students are appropriately trained for the labour market as soon as they graduate (the so-called Day-1 competences). Animal welfare Day-1 competences were also assessed and evaluated.

The results of our survey showed that the teaching of AWSEL has increased across the board, meaning that either European VEEs have responded to the growing societal needs and concerns pertaining to animal welfare and/or that welfare is more and more internally embedded in the profession, and that this is reflected in the curriculum. In terms of coverage of the Day-1 competencies, the results represent a substantial improvement from those in 2013, when more than half (55.2%) of VEEs had not met the Day-1 competences. At that time, 63.6% of the 55.2% VEEs not yet meeting the Day-1 competences thought they could meet them within the next 5 years, which is in line with the results from the present survey. In 2019, around one quarter of the 59% of European VEEs that answered the survey still only partially met the 2013 Day-1 competencies, and no information was obtained from 41% of the Veterinary Schools in Europe. This indicates the need for further efforts, both from the EAEVE and individual veterinary schools, to ensure that all Day-1 competences in the field of AWSEL are included in the curriculum as soon as possible.

In the teaching of animal welfare, ethics fares worse than animal welfare science and law, since 37% of VEEs only partially met or did not meet the Day-1 competencies. It is not surprising that animal welfare science, which has evolved substantially over the years, is more emphasised than the teaching of ethics [8]. A qualitative study at three European VEEs showed that ethics in veterinary education is a complex, multidimensional subject, composed of a range of teaching topics that might not seem to be closely connected to veterinary science [12]. This may make it difficult to explicitly define the teaching of animal welfare ethics, as the views for which course contents are best suited for veterinary students might differ. Furthermore, incorporating ethics into the education of health professionals often involves attitudinal competencies, namely through the transmission of desirable moral behaviours, or of decision-making skills, which require student-centred approaches to teaching [13]. The diversity of values regarding the moral status of animals, within and amongst European countries, and therefore differences in specific (not European) national regulations (e.g., bullfighting, gavaging geese for foie gras) may represent additional challenges to the establishment of a common curriculum in animal welfare ethics and law.

Reviewing the Day-1 competences, the vast majority of respondents found that those adopted in 2013 were still valid today, although some suggestions for improvements were made. It was deemed to be important that the differing views of stakeholders should be more prominently acknowledged. The use of ethical frameworks and theories, referred to as “ethical views on animals” in the 2013 model curriculum (point 25), was also emphasised, as it can help to reconcile veterinarians’ primary duty to safeguard the welfare of animals under their care while taking into consideration the views of society and the relevant stakeholders (e.g., farmers, animal owners). Secondly, the promotion of positive states of animal welfare, i.e., increasing quality of life through positive affective experiences [14], was seen as a topic of utmost importance. This was reflected in the increasing number of VEEs who covered this topic in their curriculum in 2019 (98%) compared with 2012 (59%). It was also felt that the amount of curriculum covering the relationship between preventive medicine (better housing, management and preventive health care) and animal welfare (in relation to reduced use of antibiotics) should be increased.

Other areas where AWSEL could contribute to more comprehensive and sympathetic veterinary contributions are shown in Table 4.

The number of obligatory and elective hours varied considerably between teaching establishments. This is not surprising, as it is often not easy to calculate the exact number of hours, as the teaching is spread over several subjects (e.g., a specific AWSEL course, veterinary public health, clinical classes, etc). Furthermore, some teaching is done in lecture style, while other faculties include practical, problem solving group seminars, group work, staged assignments or self-study. Animal welfare electives were only available in about half of the faculties (N = 30). While not possible in this study, it would have been interesting to compare the number of AWSEL teaching hours with the hours comprising the global curriculum, or relative to those dedicated to other subjects.

The basic skills and knowledge required to become a veterinarian have increased over the past five decades, with an increased focus on social skills and problem-oriented learning [15]; therefore, finding the time to teach AWSEL within the curriculum is not easy. This is not only true for Europe, but also for other parts of the world [16,17]. It is important that sufficient weight be attached to AWSEL subjects, and that they are not just taught, but also that students’ knowledge is assessed, to ensure that they are a meaningful part of the Day 1 competences [18]. Core subjects carry the advantage of being formally evaluated, whereas electives, although they can be very stimulating, may not convey the importance of the breadth of knowledge that is needed. In addition, areas of ethics to be applied in the daily execution of veterinary activities should be inculcated throughout the preclinical and clinical years. One cannot practise good veterinary medicine without practising good ethics and law. As reported in one publication, “*insufficiently mature ethical reasoning or a lack of veterinary ethical sensitivity can lead to an inability or difficulty in speaking up about concerns with clients and ultimately, failure in their advice to clients and in their duty of care to animals, leading to poor animal welfare outcomes*” [19].

The FVE European Veterinary Survey 2018 showed that veterinarians across Europe broadly agreed that the future development and sustainability of the animal sector depends upon animal welfare, under the influence of societal pressure [20]. The greater importance of animal welfare education, compared with other key veterinary subjects, in European VEEs (cf. Figure 3) mirrors the concerns of European veterinarians regarding animal welfare as a prominent future veterinary challenge. More and more specific functions require dedicated animal welfare knowledge and training, such as every slaughterhouse requiring, according to EU legislation, a dedicated Animal Welfare Officer [21], research establishments needing a ‘designated veterinarian’ to carry out animal experiments [22], and dedicated animal welfare training for official veterinary officers [10]. The European Commission ‘Farm to Fork strategy’ calls for a shift towards more sustainable food systems, bringing environmental, health and social benefits, including better animal welfare, to improve animal health and food quality, to reduce the need for medication, and to preserve biodiversity. “*It is also clear that citizens want this*.”, the strategy says [23].

Common and shared animal welfare undergraduate curricula have also been suggested by North-American, Australian and New Zealand veterinary schools [24,25]. The World Organisation for Animal Health (OIE) [10] and the animal welfare NGO World Animal Protection [26] have long promoted the teaching of animal welfare. As with many other areas of veterinary education, there is an opportunity to gain further knowledge through specialisation and postgraduate qualifications. This can also be done through specialised study at the European College of Animal Welfare and Behavioural Medicine (ECAWBM). The AWSEL speciality was recognised in 2011 by the European Board of Veterinary Specialisation (EBVS) and has grown to, at present, more than 120 veterinary specialists [27]. Similar veterinary specialisation colleges exist in North-America [28], Australia and New Zealand. This is important, as AWSEL is a global common good, and animals and animal products are increasingly moved around the world.

Some limitations of the current study should be mentioned. The response rate, although relatively high for a web-survey, may reflect the views of those schools that are more proactive in promoting the teaching of AWSEL. In addition, the limited overlap between the schools who replied to both 2012 and 2019 surveys hindered more meaningful comparisons that would have provided a picture of the evolution of AWSEL teaching at the national or regional levels. The anonymity requirements also prevented a more detailed description of participant schools. Hence, the picture of AWSEL European veterinary education presented here may not be totally accurate. Finally, the survey was designed to gather evidence from educators and deans, but its results do not shed light on the actual AWSEL competencies acquired by students, or on their opinions regarding the teaching of AWSEL at their faculties. Additional in-depth educational studies at a regional level, and also aimed at students, are therefore warranted.

## 5. Conclusions

In order to achieve the ambitious goals outlined above in the future Farm to Fork Strategy and UN SDGs, it will be necessary to devote a mixture of approaches to embed these concepts in the minds of students and to give sufficient weight to teaching AWSEL. It is, therefore, encouraging that the results from this survey show that VEEs in Europe have started to respond to these challenges by increasing the teaching of animal welfare science, ethics and law in the last 7 years, thus warranting that VEEs continue to allocate adequate time and resources to these societal important subjects.

## Figures and Tables

**Table 1:** EAEVE/FVE model animal welfare curriculum adopted in 2013.

Syllabus	Learning Objectives *(Being Able to…)*	Day One Competences
**1. Animal welfare concepts**: e.g., Biological functioning (fitness); ‘natural living’; emotional (affective) states **Analytical frameworks**: e.g., Five Freedoms, Three Rs; Concept of Quality of a Life, Ethical Matrix	1. Define animal welfare concepts 2. Recognise different welfare concepts and how they are used 3. Describe quality of life in a continuum between negative and positive welfare and overall balance 4. Analyse the scientific bases as well as the value judgements underlying each concept 5. Employ concepts in practice	1. Appraise concepts and frameworks of animal welfare
**2. Biological functioning** Adaptive capacity Motivation and cognition Sentience (feelings e.g., pain, emotions e.g., boredom, pleasure) Qualitative/quantitative assessment of internal states Stress (distress, eustress i.e., stressors to which an animal can adapt or cope) Behaviour Ethological methods Objective observation and recording Genetic/environmental interactions	6. Recognise species-specific behaviour at individual and group levels, and influence of environment and early experiences 7. Define homeostasis/allostasis 8. Distinguish between normal and abnormal behaviour 9. Describe interactions between genetics and environment 10. Appraise animals’ environmental ‘needs/wants’ and consequences of not providing them 11. Recognise the role of productivity in assessment	2. Evaluate the biological basis of welfare requirements
**3. Welfare assessment in practice** Welfare records, protocols and assurance programmes Techniques for welfare assessment Risk assessment methodology for animal welfare Housing and husbandry (including Handling and restraining of animals Transport Moving animals between environments (e.g., showing, sport and competition, conservation) Humane slaughter and killing	12. Outline the functioning of scoring systems, protocols and assurance programmes 13. Identify physiological, production and behavioural measures of welfare 14. Determine welfare hazards, exposures, consequences and risk 15. Implement basic AW records 16. Determine the welfare impact on the quality of animal products	3. Apply an animal welfare assessment to various categories of animals
**4. Personal and professional competences / attributes** Validity of scientific data Recognition of different beliefs, ethical dilemmas and moral conflicts Communication skills Professional behaviour in clinical decision making (e.g., client and animal “best interests”) Objective advice for responsible stewardship and ownership Some understanding of the philosophy of science and epistemology	17. Retrieve and make use of relevant academic literature 18. Reflect on the individual’s emotions and moral intuitions regarding animal welfare (e.g., empathy) 19. Differentiate between personal and professional standpoints 20. Appraise regulatory requirements and ethical duties 21. Promote positive welfare and minimise negative practices (e.g., pain management) 22. Recognise the role of veterinarians as educators as well as communicators 23. Communicate relevant information effectively and mediate interests	4. Formulate and communicate an informed view on animal welfare matters
**5. Human-animal relationships** Concept of duty of care Human wellbeing and animal welfare (e.g., links between animal and human abuse, burnout, suicide) Human-Animal bond Reasons for killing (e.g., slaughter euthanasia, culling, population control) Moral reasoning and ethical theories Political contexts Cultural differences Influence of economics	24. Recognise the diversity of functions and uses of animals within society 25. Describe and debate the different ethical views on animals. 26. Identify ethical dilemmas and deal with human wellbeing within the profession 27. Examine the underlying values that justify the rules and norms regarding animal welfare and protection 28. Recognise and report possible abuse of animals	5. Place animal welfare in societal and ethical contexts
**6. Welfare legislation, regulations and norms** Animal welfare regulations (regional, national, European and global) Limitations of legislation Professional standards Veterinary roles as an enforcement officer and as expert witness Veterinary certification requirements	29. Identify national, international, EU animal welfare legislation and guidelines, and OIE standards 30. Recognise animal welfare implications on trade of animals and products 31. Employ procedural guidelines, and codes of practice regarding animal welfare 32. Distinguish between formal (legal) vs. ethical responsibilities regarding the welfare of animals 33. Apply ‘private’ animal welfare standards 34. Write reports and produce satisfactory certificates	6. Place animal welfare in legal and professional contexts

**Table 2:** List of Veterinary Education Establishments that participated in the survey.

**Belgium (2/2 VEEs replied–Antwerp only teaches first years)**
University of Ghent, Faculty of Veterinary Medicine *
University of Liège, Faculty of Veterinary Medicine
University of Antwerp, Faculty of Pharmaceutical, Biomedical and Veterinary Sciences
**Bosnia and Herzegovina (1/1 VEEs replied)**
University of Sarajevo, Faculty of Veterinary Medicine
**Croatia (1/1 VEEs replied)**
University of Zagreb, Faculty of Veterinary Medicine *
**Czech Republic (1/1 VEEs replied)**
University of Veterinary and Pharmaceutical Sciences Brno, Faculty of Veterinary Medicine
**Denmark (1/1 VEEs replied)**
University of Copenhagen, School of Veterinary Medicine and Animal Science *
**Estonia (1/1 VEEs replied)**
Estonian University of Life Sciences *
**Finland (1/1 VEEs replied)**
University of Helsinki, Faculty of Veterinary Medicine *
**France (3/4 VEEs replied)**
National Veterinary School of Toulouse
Oniris Veterinary Medicine School, Nantes
National Veterinary School of Lyon (VetAgro Sup-Campus Vétérinaire)
**Germany (5/5 VEEs replied)**
Freie Universität Berlin, Department of Veterinary Medicine
Justus-Liebig-Universität Gießen, Department of Veterinary Medicine
Leipzig University, Faculty of Veterinary Medicine
University of Veterinary Medicine Hannover
Ludwig-Maximilians-University Munich, Faculty of Veterinary Medicine *
**Greece (1/2 VEEs replied)**
Aristotle University of Thessaloniki, Faculty of Veterinary Medicine
**Ireland (1/1 VEEs replied)**
University College Dublin, UCD School of Veterinary Medicine
**Italy (7/13 VEEs replied)**
University of Pisa, Department of Veterinary Sciences
University of Perugia, Department of Veterinary Medicine
University of Bologna, Department of Veterinary Medical Sciences
University of Camerino, School of Biosciences and Veterinary Medicine
University of Milan, Faculty of Veterinary Medicine *
University of Padova, Department of Animal Medicine, Production and Health
University of Messina, Department of Veterinary Science
**Lithuania (1/1 VEEs replied)**
Lithuanian University of Health Sciences, Faculty of Veterinary Medicine
**North Macedonia (1/1 VEEs replied)**
Ss. Cyril and Methodius University in Skopje, Faculty of Veterinary Medicine *
**Norway (1/1 VEEs replied)**
Norwegian University of Life Sciences, Faculty of Veterinary Medicine
**Poland (4/4 VEEs replied)**
University of Life Sciences Lublin, Faculty of Veterinary Medicine
University of Warmia and Mazury Olsztyn, Faculty of Veterinary Medicine
Warsaw University of Life Sciences, Faculty of Veterinary Medicine *
Wroclaw University of Environmental and Life Sciences, Faculty of Veterinary medicine *
**Portugal (3/6 VEEs replied)**
School University Vasco da Gama, Faculty of veterinary Medicine
University of Lisbon, Faculty Veterinary Medicine
University of Évora, Department of Veterinary Medicine
**Romania (1/4 VEEs replied)**
University of Agricultural Sciences and Veterinary Medicine Ion Ionescu de la Brad (Universitatea de Științe Agricole și Medicină Veterinară “Ion Ionescu de la Brad” Iași)
**Russian Federation (1/5 VEEs replied)**
Stavropol State Agrarian University, Faculty of Veterinary Medicine
**Spain (6/11 VEEs replied)**
Cardenal Herrera University (CEU), School of Veterinary Medicine
Complutense University of Madrid, Faculty of Veterinary Medicine
University of Extremadura, Faculty of Veterinary Medicine
Autonomous University of Barcelona, School of Veterinary Science *
University of Córdoba, Faculty of Veterinary Science
University of León, Faculty of Veterinary Medicine
**Sweden (1/1 VEEs replied)**
Swedish University of Agricultural Sciences (SLU), Faculty of Veterinary Medicine and Animal Science (VH) *
**Switzerland (1/1 VEEs replied)**
University of Zurich, Vetsuisse Faculty
**The Netherlands (1/1 VEEs replied)**
University of Utrecht, Faculty of Veterinary Medicine
**Turkey (5/13 VEEs replied)**
Ankara University, Faculty of Veterinary Medicine
Aydin Adnan Menderes University, Faculty of Veterinary Medicine
Bursa Uludag University, Faculty of Veterinary Medicine
Selcuk University, Faculty of Veterinary Medicine
Firat University, Veterinary Medical School
**United Kingdom (5/7 VEEs replied)**
University of Cambridge, Department of Veterinary Medicine *
Royal Veterinary College, London
University of Bristol, Bristol Veterinary School *
University of Edinburgh, College of Medicine & Veterinary Medicine
University of Nottingham, School of Veterinary Medicine and Science

* Schools that had also answered the 2012 survey.

**Table 3:** Teaching of specific animal welfare topics.

Topic	Yes, this Subject is as a Whole or Partly Covered		I don’t Know		No, this Subject is not Covered	
	2019	2012	2019	2012	2019	2012
Basic and applied ethology	96%	85%	4%	0%	0%	15%
Stress physiology	91%	88%	7%	6%	2%	6%
Animal Welfare Assessment Farm animals	95%	79%	2%	0%	4%	21%
Animal Welfare Assessment Companion animals	89%	66%	7%	6%	4%	28%
Animals Welfare Assessment Laboratory animals	75%	65%	12%	6%	12%	29%
Animals Welfare Assessment Zoo animals	51%	24%	16%	15%	33%	62%
Animals Welfare Assessment Wild animals	54%	29%	18%	12%	28%	59%
Legal issues concerning AW	91%	94%	9%	0%	0%	6%
Animal ethics	88%	82%	11%	6%	2%	12%
Professional veterinary Ethics & Etiquette	93%	85%	5%	3%	2%	12%
Principles disease prevention	98%	94%	2%	0%	0%	6%
The promotion of positive animal welfare	98%	59%	0%	15%	2%	26%
Handling and restraining of animals	98%	91%	0%	3%	2%	6%
Assessing and controlling pain	95%	82%	4%	3%	2%	15%
Welfare around euthanasia	93%	85%	7%	9%	0%	6%
Assess and implement basic welfare records	88%	59%	5%	21%	7%	21%

**Table 4:** Other areas where respondents indicated that a veterinary contribution could be made.

1. Emphasising integrating statistics and good scientific practices in animal research which should, ideally, be carried out scientifically. (See also the PREPARE and ARRIVE guidelines in animal research for other applicable guidance.)
2. Emphasising integrating better scientific practices in animal protection which should, ideally, be carried out sympathetically and incorporate a better understanding of human behaviour and attitudes.
3. Teaching more on the biological functioning of the animal species involved, their behavioural repertoire and motivations, behavioural development, and a clinical approach to behavioural disorders so that animal suffering can be mitigated.
4. Incorporating the concept of “animal expectations” (see ANSES 2018) and its application.
5. Including the application of the Three Rs ethical framework in areas of animal use other than in experimentation.
6. Integrating AWSEL more into clinical veterinary ethical dilemmas in practice, such as the integration of a better understanding of animal behaviour e.g., low-stress handling, pain recognition, euthanasia, overtreatment, clinical research, etc. These clinical aspects are important in engaging veterinarians in the ‘real-world’ applications of AWSEL.
7. Increasing the teaching of AWSEL e.g., scientific approaches to the assessment of avoidable animal pain, distress and suffering in all aspects of animal use globally. There is enormous potential for this approach to have a wide impact in the future.

## Appendix A. Survey Questions 2019

1. Contact details (First name, Last name, E-mail address, Name of your University, School or Organisation, Country, Position or area of responsibility)
2. Considering Animal Welfare Science, Ethics and Law education in undergraduate veterinary studies, do you believe the level of education in your teaching establishment has in the last 6 years Increased substantially/Increased/Stayed the same/Decreased
3. Are Animal Welfare Science, Ethics and Law education part of the core obligatory curriculum? I. e. students have an exam and can theoretically fail in the same way as other core subjects?
4. Looking at the 2013 Model Curriculum above, do you think it still covers the important learning objectives and Day-1 competences in animal welfare today? (Yes-certainly, Yes, No, No-certainly not, Unknown) + possibility free text
5. Looking at the 2013 model curriculum above and looking at the establishment where you teach, do you think that for Animal Welfare Science, Ethics and Law education they are covering these Day-1 competences today? (Exceeding the Day-1 competences, Meeting the Day-1 competences, Meeting partly the Day-1 competences, Far from meeting the Day-1 competences, Unknown) + possibility free text
6. If you are not already reaching the learning outcomes, in how many years do you think you could do so (give number of years or unknown)
7. If you are not already reaching the Day-1 competences, to what extent (Completely, Mainly, Amongst other reasons, This is not the problem, Unknown) is any delay due to (Lack of qualified teachers in any particular subject, Lack of space in the curricula, Financial reasons) + possibility free text
8. Is animal welfare a study topic in the same way as other subjects, such as surgery or radiology at your college? (Yes, No-not emphasised in other subjects, No-emphasised as part of other subjects) + possibility free text
9. Is animal welfare offered as an elective or as an area of mentored independent study? Please indicate below, and explain yourself if animal welfare is part of another clinical rotation. (Yes-specific elective, Yes-mentored independent study, Part of other elective) + possibility free text
10. How many obligatory and elective hours does the undergraduate veterinary syllabus allocate to the study of Animal welfare Science, Ethics and Law?
11. Please rank the importance to veterinary medical education of the following contemporary issues associated with animals (1 = most important and 5 = least important issue): Food safety, Conservation (wild species & local breeds), Intensive agriculture, Animal welfare, Biotechnology). Please rank the importance to veterinary medical education of the 2 following issues associated with animals (1 = most important and 5 = least important issue): Animal welfare, Antimicrobial resistance
12. How important is students’ knowledge of Animal Welfare Science, Ethics & Law to your veterinary college? (Very important, Somewhat important, Not important, Unsure)
13. How important do you think it is that there is a course called Animal Welfare in the veterinary undergraduate syllabus? (Very important, A bit important, Irrelevant, Not important at all) + possibility free text
14. Looking at your present curriculum please indicate whether the following subjects are day 1 skills? (Basic and applied ethology, Stress physiology, Animal Welfare Assessment of Farm animals, Animal Welfare Assessment of Companion animals, Animal Welfare Assessment of Laboratory animals, Animal Welfare Assessment of Zoo animals, Animal Welfare Assessment of Wild animals, Legal issues concerning animal welfare, Animal welfare ethics, Professional veterinary Ethics and Etiquette, The principles of disease prevention and promotion of health, The promotion of positive animal welfare, Handling and restraining of animals, Assessing and controlling pain, Recognise when euthanasia is necessary and perform it humanely, Assess and implement basic welfare records (e.g., Welfare Quality protocols)) they are covered (Yes, this subject is covered, Parts of this subject are covered, I don’t know, No, this subject is not covered).
